# A study of association between expression of hOGG1, VDAC1, HK-2 and cervical carcinoma

**DOI:** 10.1186/1756-9966-29-129

**Published:** 2010-09-17

**Authors:** Peng Guo-Qing, Yang Yuan, Zhong Cai-Gao, Yin Hongling, Hu Gonghua, Tian Yan

**Affiliations:** 1 Department of O & G, Xiangya Hospital Central-South University, Changsha Hunan 410008, China; 2 Department of Clinical Laboratory, Huaihua Medical College, Huaihua Hunan 418000, China; 3 School of Public Health, Central South University, Changsha Hunan 410008, China; 4 Department of Pathology, Xiangya Hospital Central-South University, Changsha Hunan 410008, China

## Abstract

**Background:**

Human 8-oguanine glycosylase 1(hOGG1), voltage-dependent anion channel 1(VDAC1), hexokinase 2(HK-2), represented the process of oxidative DNA damage, cell apoptosis and glycolysis, respectively. This study aims to explore the association between expression of hOGG1, VDAC1, HK-2 and cervical carcinoma.

**Methods:**

A case-control study was conducted. 65 cervical biopsy samples consist of 20 control and 45 cases. The expression of hOGG1, VDAC1 and HK-2 were examined with immunohistochemistry(IHC), immunolabeling was evaluated with stereological cell counts.

**Results:**

The data showed that the positive proportion of hOGG1 and HK-2 in the case group was higher than that of the control group (P < 0.05). Further, there was an increasing trend for the positive proportion and expression degree of hOGG1 and HK-2 from Control, Mild cervical carcinoma (MCC), Intermediate cervical carcinoma(ICC) to Severe cervical carcinoma(SCC) in order (P < 0.05). To VDAC1, the significant result was not obtained.

**Conclusions:**

The results suggested that there was a close association between expression of hOGG1, HK-2 and cervical cancer. hOGG1 and HK-2 might play a key role at the early stage of cervical cancer, and the findings of hOGG1 and HK-2 should be considered as a significant biomarker at the early stage of cervical cancer.

## Background

Cervical cancer is currently one of the most frequently occurring cancer among women[1]. In China, Sample surveys showed that Cervical cancer is the major cause of death in women, the proportion of death rank in the fourth place, only behind gastric carcinoma, esophageal carcinoma, hepatic carcinoma[2]. Furthermore, the age range of cervical cancer incidence become more and more younger since the past 30 years[3-5]. At the present, researchers considered cervical cancer as a disease which is impacted by many factors, and these factors was classified as environment cause or genetic factors, Such as infection of human papilloma virus(HPV) and human immunodeficiency virus(HIV), ill behavior of sex, smoking, chromosome deficiency, Single Nucleotide Polymorphism(SNP), etc[6-8]. Prevention of cervical cancer is still an unsettled puzzle. At the present, early-stage cervical cancer could be detected mainly by cytological screening of papanicolaou smear test and pathological diagnosis of cervical biopsy sampling. To cervical cancer, the mainly method of therapy were still surgical, chemical and radialion therapy. The result of treatment depended on early discovering of cervical carcinoma in great degree.

In recent study, some abnormal molecular biology changes are considered playing a central role in process of cervical cancer and cervical precancerous lesion. And these biomarkers of abnormal molecule can be used to forecast the incidence probability of cervical precancerous lesions. Consequently, the patient condition of early discovering will be improved obviously through earlier therapy. In recent years, many significant study findings were obtained, for example, study of Reddy VG et al[9,10]showed that telomerase activity was detected in 96.5% of cervical tumor samples and in 68.7% of premalignant cervical scrapings but was not detected in control hysterectomy samples and in cervical scrapings of normal healthy controls. The absence of telomerase activity in cervical scrapes from healthy women indicated the potential of telomerase to serve as a good screening marker for the early diagnosis of cervical cancer. Murphy N et al[11,12] found that p16INK4A expression was closely associated with high risk HPV infection, all grades of squamous and glandular cervical lesions were IHC positive, p16INK4A was the most reliable marker of cervical dysplasia.

Generally, oxidative DNA damage, cell apoptosis, glycolysis were considered playing a essential role in the dynamic process of neoplasm. Many environmental factors could induce production of oxidative DNA damage, and further continual evolution, the following result was genetic mutation, dysfunction of cell cycle, apoptosis. Majority of normal cell died in the form of apoptosis, and minority of abnormal cell survived yet and grew unlimited. Ultimately, abnormal cell is stimulated and activated in the form of neoplasm cell. Furthermore, Its mainly mode of energy production was glycolysis metabolism[13-15]. Our current question is, did the similar physiological course of malignant transformation occur also in the transformation process from normal cervical tissue to cervical cancer? At present, relatively study is documented rarely about the combined feature of oxidative DNA damage, cell apoptosis, glycolysis in cervical cancer tissue. Therefore, we selected three genes[16-18], Human 8-oguanine Glycosylase 1(hOGG1), voltage-dependent anion channel 1(VDAC1), hexokinase 2(HK-2), represented the process of oxidative DNA damage, cell apoptosis, glycolysis, respectively. And the expression of hOGG1, VDAC1, HK-2 were detected by the method of IHC for exploring the association between them and cervical cancer.

## Materials and methods

### Tissues samples

65 paraffin wax-embedded cervical biopsy samples were selected from the pathology department of the Xiangya Hospital, Central-South University. These samples were divided into two groups containing 20 control and 45 cases, and 45 cases of cervical cancer including 15 mild, 17 intermediate, 13 severe according to pathological diagnosis. Haematoxylin and eosin stained slides of all biopsy samples were reviewed by two pathologists and classified according to criteria outlined by the World Health Organization (WHO). Ethical approval for use of all specimens was obtained from the research ethics committee of the Xiangya Hospital.

### Antibodies

Available Rabbit anti-Human polyclonal antibody HK-2 was from Abnova, USA; 8-oxoguanine DNA Glycosylase Homolog 1 (OGG1) and Voltage-Dependent Anion Channel 1 (VDAC1) Rabbit anti-Human Polyclonal Antibody were all from LifeSpan BioSciences, USA.

### IHC on biopsy samples

Sections (4 μm thick) were cut from paraffin wax embedded biopsy samples and mounted on 3-aminoproplytriethoxysilane coated glass slides. Sections were dewaxed by passage through xylene and then rehydrated in graded alcohol. Endogenous peroxidase activity was blocked by incubating the sections in 3% H_2_O_2 _for 10 minutes. Antigen retrieval was performed in 0.01 M citrate buffer (pH 6.0) using high pressure cooker for 15 minutes. After washing sections in Phosphate Buffered Saline(PBS, pH 7.4), non-specific antibody binding was reduced by incubating the sections in 10% normal goat serum for 15 minutes. After decanting excess serum, sections were incubated overnight at 4°C with primary rabbit anti-human polyclonal antibody HK-2 (1:50 dilution), OGG1 (1:100 dilution), or VDAC1 (1:500 dilution). Sections were washed three times for 5 minutes at the following day, respectively. Adding polymer enhancer 50 ul and incubating for 20 minutes, repeating previous washing method. After washing thoroughly with PBS, the sections were incubated for 20 minutes with secondary antibody horseradish peroxidase(HRP)-polymer anti-goat IgG at room temperature. The avidin-peroxidase protocol (ABC Kit-5020; Abnova) was applied in the last step of the procedure, using 3, 3-diaminobenzidine(Sigma, St. Louis, MO, USA) as chromogen. The sections were counterstained lightly with haematoxylin. Finally, the sections were dehydrated, cleared, coverslipped. Controls were carried out with the same protocols but omitting the primary antibodies, which did not result in any staining.

### Statistical analysis

The results of experiment was collected by computer, the process of data analysis was carried out by Microsoft office Excel 2003 and SPSS13.0. The Pearson Chi-Square (*χ^2^*) test was used to compare difference between two groups. The development trend of CIN was evaluated by the method of *Linear χ^2 ^*test. The *McNemar χ^2 ^*and Kappa statistic were used to analyze consistency level between hOGG1 and VDAC1 or HK-2. A 0.05 *P*-value of two-sided test was the standard of statistics significant. For the sake of statistical convenience, the positive results of ±,+,++ and +++ were merged into one group.

## Results

### IHC staining of hOGG1, VDAC1, HK-2

**Figure 1:**
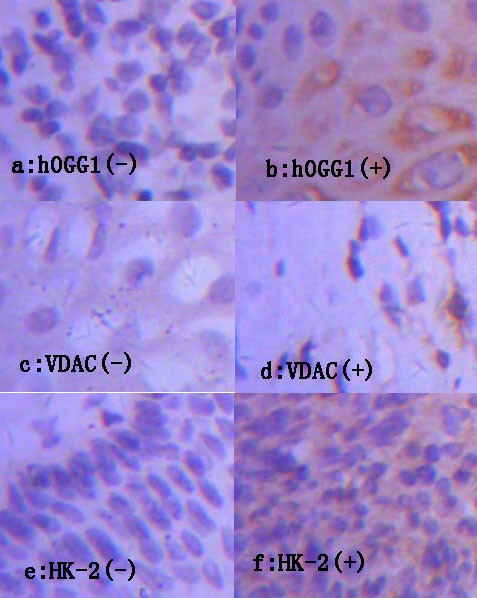
**The expression of hOGG1, VDAC1, HK-2 was displayed by figure a,b,c,d,e,f in turn, figure a,c,e were representative of negative expression, while figure b,d,f were indicative of positive expression, respectively**.

All staining sections were conserved in the form of pictures. The pictures showed that hOGG1 and HK-2 located in cervical epithelial tissue or glands or cytoplasm of cervical biopsy samples, VDAC1 located in cervical epithelial tissue or glands or cell membrane of cervical biopsy samples. The positive result of staining was yellow or brown yellow. The map of expression of hOGG1, VDAC1, HK-2 was listed partially (Figure 1). The result of positive or negative was diagnosed by the method of stereological cell counts. The absence of positive cell was indicative of negative(-). when observed positive cell was less than 25 percent, the result of diagnosis was slightly positive(±). when the proportion of positive cell ranged from 25 to 50 Percent, the result of diagnosis was positive(+). When more than 50 percent of positive cell was observed, we considered it intense positive (++).

### Expression of hOGG1, VDAC1, HK-2 in cervical biopsy samples

**Figure 2:**
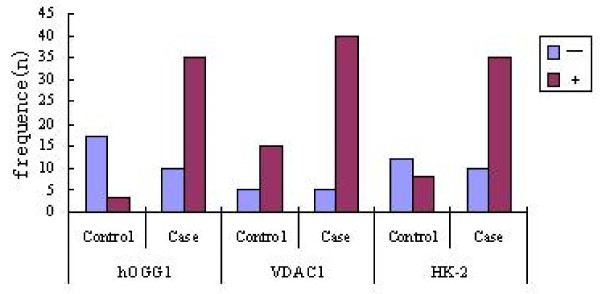
**Distribution of expression of hOGG1, VDAC1, HK-2 in control and case**.

We compared the positive proportion of hOGG1, VDAC1, HK-2 between control and case group, the table 1 showed that there were a significant difference for hOGG1, HK-2 between two groups. To VDAC1, the difference was not obviously significant. More detail in Table 1 and Figure 2.

**Table 1:** Distribution of expression of hOGG1, VDAC1, HK-2 in control and case

groups	n	hOGG1			VDAC1			HK-2		
		
		-	+	+%	-	+	+%	-	+	+%
Control	20	17	3	15.0	5	15	75.0	12	8	40.0
Case	45	10	35	77.8	5	40	88.9	10	35	77.8
total	65	27	38	58.5	10	55	84.6	22	43	66.2

*χ^2^*		22.47			1.12			8.83		
*P*		0.000			0.289			0.003		

Note: *P *value of *χ^2 ^*is indicative of result of comparison between control and Cases, When *P *value is below standard of 0.05, the level of difference is significant.

**Figure 3:**
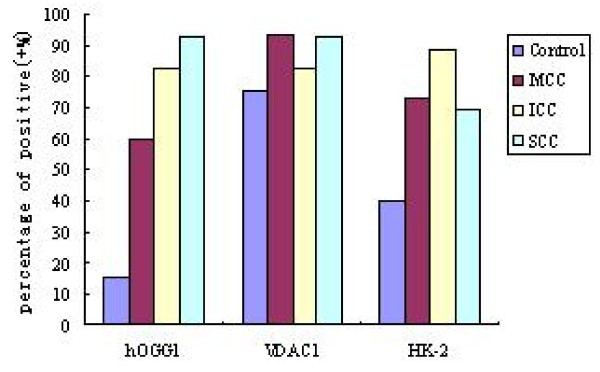
**Expression of hOGG1, VDAC1, HK-2 in classified cervical biopsy samples**.

Further, we analyzed the divided Cases samples according to pathology diagnosis for more valuable information. As described in the Table 2, Figure 3, the proportion of positive expression of hOGG1 and HK-2 showed an increasing trend from Control, MCC, ICC to SCC in order. To VDAC1, the increasing trend of positive proportion was not observed.

**Table 2:** Expression of hOGG1, VDAC1, HK-2 in classified cervical biopsy samples

	hOGG1			VDAC1			HK-2		
	
	-	+	+%	-	+	+%	-	+	+%
Control	17	3	15.0	5	15	75.0	12	8	40.0
MCC	6	9	60.0	1	14	93.3	4	11	73.3
ICC	3	14	82.4	3	14	82.4	2	15	88.2
SCC	1	12	92.3	1	12	92.3	4	9	69.2

*Linear χ^2^*	23.295			1.171			5.207		
*P*	0.000			0.279			0.023		

Note: MCC, ICC and SCC were explained in abbreviations, *Linear χ^2 ^*was used to analyze trend of expression from control, MCC, ICC to SCC in order. When *P *< 0.05, the trend is significant.

### Comparison of consistency level of hOGG1, VDAC1 and HK-2

In order to observe the consistency of expression hOGG1, VDAC1 and HK-2, Pair *χ^2 ^*test and Kappa value was used to analyze the consistency level of three pairs of hOGG1--VDAC1, hOGG1--HK-2, VDAC--HK-2. As showed in the Table 3, Overall, there was a low level of consistency expression in pairs of hOGG1--VDAC1, VDAC--HK-2 and hOGG1--HK-2.

**Table 3:** The consistency level of expression of hOGG1, VDAC1, HK-2 in cervical samples

		VDAC1		HK-2				HK-2	
		-	+	-	+			-	+

hOGG1	-	5	22	14	13	VDAC1	-	5	4
	+	5	33	8	30		+	17	39
*χ^2^*	9.48			0.76			6.86		
*P*	0.002			0.383			0.007		
*Kappa*	0.059			0.316			0.157		

Note: Pair *χ^2 ^*or *McNemarχ^2 ^*is used to analyze consistency level commonly, When *P *< 0.05, the consistency level is not significant. Similarly, When *Kappa *< 0.45, indicating a low consistency level.

### Relationship between expression degree of hOGG1, VDAC1, HK-2 and classified cervical biopsy samples

65 cervical biopsy samples were classified as -, ±, + and ++ four types or Control, MCC, ICC and SCC four groups according to proportion of positive cell or pathology diagnosis. As a result, we observed that relationship between expression of hOGG1, VDAC1, HK-2 and graded pathology types of cervical biopsy tissue. As showed in Table 4, there was an increasing trend for the expression degree of hOGG1 and HK-2 from Control, MCC, ICC to SCC in order. To VDAC1, This trend of statistical significance was not observed.

**Table 4:** Relationship between expression degree of hOGG1, VDAC1, HK-2 and pathology types

	hOGG1				VDAC1				HK-2			
	-	±	+	++	-	±	+	++	-	±	+	++

Control	17	3	0	0	5	1	10	4	12	4	4	0
MCC	6	5	4	0	1	0	7	7	4	5	4	2
ICC	3	0	7	7	3	3	7	4	2	5	9	1
SCC	1	1	7	4	1	3	7	2	4	3	5	1

*χ^2^*	33.54				0.049				8.358			
*P*	0.000				0.825				0.004			

Note: The *χ^2 ^*was used to analyze the bidirectional trend of hOGG1, VDAC1 and HK-2, When *P *< 0.05, the trend was significant.

## Discussion

Cervical cancer is the secondary frequently occurring carcinoma among women. Its incidence rate is from 3.25-10.28 per 100000 approximately in china, lower only than breast neoplasm[8]. Generally, people consider that cervical cancer is a disease activated by many factors, the dynamic mechanism of Cervical cancer is not yet elucidated completely due to the complexity of pathogeny evolvement pathway. In the same way, the screening of early and sensitive biomarker is also an unsettled problem. Furthermore, cervical cancer is associated closely with oxidative DNA damage, cell apoptosis, glycolysis. To explore the unsettled puzzle, develop more significant biomarker of cervical cancer and cervical precancerous lesions, we analyzed the expression of hOGG1, VDAC1 and HK-2 in cervical biopsy tissue. The following result was exhibited orderly.

① The result of experiment showed that the positive proportion of hOGG1 and HK-2 in the case group was higher than that of the control group (P < 0.05), there was no obvious differentiation for positive proportion of VDAC1 in the case group and the control group; ② Further, statistical analysis showed that there was an increasing trend for the positive proportion of hOGG1 and HK-2 from Control, MCC, ICC to SCC in order. To VDAC1, the increasing trend of positive proportion was not observed; ③ Consistent pair study showed that there were a lowly level of consistency expression in pairs of hOGG1--VDAC1, VDAC1--HK-2 and hOGG1--HK-2. The range of Kappa value was from 0.059 to 0.316. The result indicated that there was no interaction effect in pairs of hOGG1--VDAC1, VDAC1--HK-2 and hOGG1--HK-2; ④ In addition, we observed that relationship between expression degree of hOGG1, VDAC1, HK-2 and graded pathology types of cervical biopsy tissue. The result indicated that there was an increasing trend for the expression degree of hOGG1 and HK-2 from Control, MCC, ICC to SCC in order. To VDAC1, the significant trend was not observed.

The above description indicated that there was close association between expression of hOGG1, HK-2 and Cervical cancer. hOGG1 was one of glycosylases in the base excision repair (BER) system, played a central role in removing adducts from oxidative DNA damage, which was nominated by 8-Oxo-7,8-dihydroguanine (8-oxoGua)[16]. When DNA repair system of the organism is normal, the expression level of hOGG1 can reflect indirectly accumulated level of 8-oxoGua in organism. Therefore, we combined the result of higher positive proportion of hOGG1 in the case group with an increasing trend for the positive proportion of hOGG1 from Control, MCC, ICC to SCC in order. We could draw a conclusion that oxidative DNA damage existed in early stage of cervical cancer, the increasing expression degree of hOGG1 reflected severity of oxidative DNA damage in the progress of cervical cancer and the precancerous lesions. Our hypothesis was that many outside factors can induce the production of irritative oxidative reaction, further, it produced excessive reactive oxygen species(ROS) and attacked cell nucleus DNA, resulting in an increasing level of accumulated 8-oxoGua. 8-oxoGua is an abnormal DNA base. Which has capacity of inducing gene mutation and neoplasm[19]. As a result, we proposed that oxidative DNA damage was probably one of dynamical mechanism of cervical cancer. The level of oxidative DNA damage can be reflected indirectly by DNA repair gene hOGG1. Therefore, maybe hOGG1 play a crucial role at early stage of cervical cancer, and detection of hOGG1 is valuable for the early discovering of cervical cancer.

Our experiment proved that HK-2 was associated with cervical cancer as well. HK-2 is one of crucial enzyme involved in the conversion of hexose phosphate in pathway of cell glycolysis. While cell be in the case of mitochondria dysfunction, glycolysis reaction is activated to produce ATP for compensating the supply of energy of cell survival and growth. But the method of through glycolysis pathway is not an effective way of ATP production, which is one condition of abnormal energy supply. As a result, it can influence normal condition of cell differentiation and Cell proliferations, and finally constitutes the underlying basis of neoplasm cell [20]. Some experiments testified that HK-2 is binding to mitochondria in carcinoma tissue, such mode of binding is helpful for HK-2 making use of energy produced by mitochondria[21]. Other study discovered also that HK-2 was adhered to outer mitochondrial membrane(OMM), and interacted with VDAC1 executing anti-apoptosis effect[22,23]. Therefore, on the one hand the expression of HK-2 could reflect level of glycolysis, on the other hand it reflected a lower level of cell death as well. Our experiment proved that the positive proportion and level of expression of HK-2 showed an increasing trend along the progress of cervical cancer. Such result indicated that energy mechanism of glycolysis existed in early stage of cervical cancer, and when cervical neoplasm progressed forward in irreversible way, level of glycolysis in cell was increasing correspondingly, and level of cell death is decreasing at the same time. As a result, we proposed considerately that HK-2 should be considered as a significant biomarker at the early stage of cervical cancer and the cervical precancerous lesions. Further, the degree of expression of HK-2 could reflect the degree of neoplasm tissue transformation malignant. In addition, the consistency experiment showed that no statistical significant interaction was observed between HK-2 and VDAC1, further study was essential for discovering more valuable information.

## Abbreviations

CIN: cervical intraepithelial neoplasia; hOGG1: human 8-oguanine glycosylase 1; VDAC1: voltage-dependent anion channel 1; HK-2: hexokinase 2; MCC: mild cervical carcinoma including CINIand CINII; ICC: intermediate cervical carcinoma implicating CINIII; SCC: Severe cervical carcinoma implicating invasive squamous carcinoma.

## Competing interests

The authors declare that they have no competing interests.

## Authors' contributions

PGQ and TY designed the study and collected the cervical biopsy samples, YY and TY wrote the main manuscript, HGH performed data analysis, YHL accomplished pathological diagnosis, ZCG looked over the manuscript. All authors read and approved the final manuscript.
