# Correction: A study of association between expression of hOGG1, VDAC1, HK-2 and cervical carcinoma

**DOI:** 10.1186/1756-9966-30-53

**Published:** 2011-05-11

**Authors:** Peng Guo-Qing, Yang Yuan, Zhong Cai-Gao, Yin Hongling, Hu Gonghua, Tian Yan

**Affiliations:** 1 Department of O & G, Xiangya Hospital Central-South University, Changsha, China; 2 School of Public Health, Central South University, Changsha, China; 3 Department of Clinical Laboratory, Huaihua Medical College, Huaihua, China; 4 Department of Pathology, Xiangya Hospital Central-South University, Changsha, China

## Correction

After publication of this work [1], we noted that we inadvertently made an error order of author affiliations. The corrected order of author affiliations was listed as above.

## Competing interests

Dr Guo-qing P and Yan T made main contribution for this works, and have consulted the other authors in competing interests. They declare no conflicts of interest.

## Authors' contributions

PGQ and TY designed the study and collected the cervical biopsy samples, YY wrote the main manuscript, HGH performed data analysis, YHL accomplished pathological diagnosis, ZCG looked over the manuscript. All authors read and approved the final manuscript.
